# Phytochemical profile, comparative evaluation of *Satureja montana* alcoholic extract for antioxidants, anti-inflammatory and molecular docking studies

**DOI:** 10.1186/s12906-023-03913-0

**Published:** 2023-04-06

**Authors:** Khaled Abdelhady Abdelshafeek, Abeer Fouad Osman, Samar Mohamed Mouneir, Ahmed Abdelmonem Elhenawy, Walid Elsayed Abdallah

**Affiliations:** 1 Chemistry of Medicinal Plants Department, Pharmaceutical and Drug Industries Research Institute, National Research Centre, 33 El Buhouth St. (Former El Tahrir St.), 12622-Dokki, Giza, Egypt; 2 Chemistry of Natural Compounds Department, Pharmaceutical and Drug Industries Research Institute, National Research Centre, 33 El Buhouth St. (Former El Tahrir St.), 12622-Dokki, Giza, Egypt; 3 Department of Pharmacology, Faculty of Veterinary Medicine, Cairo University, Cairo, 12211 Egypt; 4 Chemistry Department, Faculty of Science, Al-Azhar University, 11884 Nasr City, Cairo, Egypt

**Keywords:** *Satureja montana*, Lamiaceae, Ursolic acid, Flavonoids, Anti-inflammatory activity, Molecular docking

## Abstract

**Background:**

The most common species in the *Satureja* genus is *Satureja montana* (family Lamiaceae). The present work aims to use the molecular docking study to predict the isolated constituents against an immune system immunomodulator and tested alcoholic extract as an in-vitro immunomodulatory agent.

**Methods:**

All isolated compound's structures were determined using various chromatographic and spectroscopic techniques. Anti-inflammatory and antioxidant profiles were studied for an alcoholic extract of the plant; the molecular docking study was performed for the isolated compounds (1–4).

**Results:**

In this work, four recognized compounds were extracted from the ethyl acetate fraction of *S. montana* (*Sm*) methanolic extract and identified as two triterpenes ursolic acid (1) and oleanolic acid (2), one phenolic acid as ellagic acid (3), and one flavonoidal compound as isoscutellarein (4).

The total alcoholic extract exhibited good in vitro anti-inflammatory, antioxidant, and apoptotic activity. Its IC_50_ was 10.12 compared to 15.1 μg/ml of standard celecoxib. It also showed potent antioxidant activity with IC_50_ 10.4, 11.3, 14.6, and 22.3 IU/ml for SOD, MDA, catalase, and TAC, respectively. According to the drug similarity and ADMET tests, their ligands may have favorable pharmacokinetic characteristics with minimal carcinogenic influence. The molecular docking study was performed for the isolated compounds (1–4).

**Conclusions:**

The alcoholic extract of the plant showed promising anti-inflammatory, antioxidant, and antiapoptotic properties. The theoretical studies for the isolated compounds showed promising binding affinity against all the examined enzymes.

## Background

The Lamiaceae family is an important medicinal plant family. It includes about 236 genera and more than 6000 species. This family has great variations which are widely distributed all over the world [1]. *Satureja* (family Lamiaceae) is a genus of over 200 aromatic herbs and shrubs endemic to the Middle East, with more than 30 species found in the eastern Mediterranean. They are utilized as culinary herbs as well as in traditional medicine to treat a wide range of ailments, like antimicrobial, spasmolytic, hepatoprotective, antiviral, and anticarcinogenic effects [2]. Also, antioxidative efficacy can affect the etiology of chronic diseases and the aging process [3]. It was reported from the previous phytochemical studies on some species of this genus, the presence of phenolic acids, flavones, anthocyanins, sterols, diterpenes, and triterpenes [4–8]. The most widespread species in this genus, *Satureja montana*, has been the focus of much research that has revealed the existence of numerous chemical components and biological activity. The existence of luteolin-7-rhamnoside-4'-*O*-glucopyranoside, quercetin-3-*O*-L-rhamnopyranoside, and quercetin7-*O*-glucopyranoside antibacterial activity against all tested microbes was demonstrated in recent research on *S. montana* by the authors [9]. Also, the previous study showed higher antioxidant activity (87.7%) for the alcoholic extract than that for the hexane fraction. Furthermore, the immunomodulator term is the substance that helps in regulating the immune system of an organism. Immunomodulators are divided into two types; those that increase the immune response (immunostimulants) and those that decrease the immune response (immuno-suppressants). Aside from the various agents that can aid in the regulation of an overactive or underactive immune system. Immunomodulators are frequently used to prevent organ rejection in autoimmune diseases such as rheumatoid arthritis and organ transplantation. In traditional systems of medicine, several plants used for vitality and long-term sickness prevention have been discovered to have immune system effects [10–12]. The present work aims to complete the isolation of other chemical constituents and biological activity of *S. montana*. Then use the molecular docking study to predict the isolated constituents against the immune system immunomodulator and tested alcoholic extract as an in-vitro immunomodulatory agent.

## Material and methods

### General experimental procedures

UV spectra were recorded on Shimadzu model UV-240 and 2401 PC spectrophotometer (Shimadzu Inc., Tokyo, Japan). NMR experiments were recorded on Bruker spectrometer (Switzerland) 600 (^1^H NMR spectra: 600 MHz; ^13^C NMR spectra: 150 MHz). The chemical shifts are given in δ (ppm) relative to tetramethylsilane (Me_4_Si) TMS. Column chromatography (CC) was carried out on silica gel, 60–200 mesh, Fluca, India) and Sephadex LH-20 (Pharmazia, Uppsala, Sweden). Paper chromatography (PC, descending) Whatman No. 1 and 3 mm papers, silica gel aluminum sheets (20 × 20) using solvent systems hexane: chloroform: methanol 90:8:2 (S-1), 15% HOAc(S-2), BAW (n-BuOH: HOAc: H_2_O) 3:1:1(S-3), and BAW 4:1:5, the upper layer (S-4).

### Plant material

*S. montana *L. was cultivated and grown on the farm of the National Research Center (NRC), Giza, Egypt, and it was collected in April 2020. The authentication of plant samples was achieved by Dr. Mohammed Elgibali a taxonomist at NRC. A voucher specimen was deposited in the herbarium of the National Research Centre (accession number SC-1510). The aerial parts were air-dried for 2 weeks under laboratory conditions at 28 ± 2 °C. The dried material was ground using a domestic blender to a fine powder [9].

### Isolation and purification of the chemical constituents

The powder of the aerial parts (2 kg) was extracted three times at room temperature with aqueous methanol (80%, 3 × 2.5 L). The methanolic extract was evaporated under reduced pressure to obtain a residue (250 g) which dissolved in hot distilled water (900 ml), and left-over night in the refrigerator and after then the precipitated matter was filtered off [9]. The aqueous filtrate was defatted with n-hexane (500 ml × 3), followed by a partition with ethyl acetate (400 ml × 3). The ethyl acetate extract (3.5 g) was chromatographed on a silica gel column (4 × 65). Elution was started up with n-hexane (2 L) with increasing the polarity by 20% stepwise addition of chloroform followed by methanol, 250 ml each were collected, and the elution was monitored using solvent S-1, the similar fractions were pooled together, and the solvents were evaporated under reduced pressure at 40^◦^C. Two main fractions were found promising, so they were subjected to further purifications as follows: Fraction -I (75 mg) eluted with hexane-chloroform (70–30) was found to contain two main compounds and further rechromatographed over a small silica gel column (1 × 25 cm) eluted with hexane-chloroform (80–20) to afford compounds 1 and 2 in pure form (18 mg and 24 mg, respectively). Fraction -II (80 mg, eluted with hexane-chloroform–methanol (50–50-10), was found to contain two main compounds (one flavonoidal compound and the other a phenolic acid) and was passed over Sephadex LH-20 column (1 × 25 cm) eluted with 100% methanol to afford compounds 3 and 4 together (Fraction -IIA). The fraction -IIA was subjected to preparative paper chromatography using Whatman 3 mm and solvent S-4 to give compounds **3** and 4 in pure form (20 mg and 25 mg, respectively).

### Molecular modeling

#### Preparation of small molecule

The 3D structures for compounds (1:ursolicacid; CHEBI:9908), (2:oleanolic acid; CHEBI:37,659), (3: ellagic acid; CHEBI:5,281,855), (4: isoscutellarein; CHEBI:66,085), were gained from NCBI web server, USA (www.pubchem.ncbi.nlm.nih.gov). The chemical structures were optimized using the PM3 semi-empirical Hamiltonian molecular orbital calculation MOPAC16 package [13], then docked to receptors as implemented in MOE 2021 package [14].

#### Selection of proteins structures

A docking experiment was carried out for the target active sites of human immunomodulatory receptors such as NF-kappa B p52 (PDB:1A3Q) [15]. Proinflammatory cytokines such as interleukin-1 “IL-1”; (PDB:2NVH), interleukin-6 “IL-6”;(PDB:1P9M) [16], antioxidant activity as (PDB:1P9M). Viral interleukin-10 (PDB:1VLK), anti-inflammatory receptors such as cyclooxygenase-2 (PDB:1CX2) [17]. MOE, 2021 [14], was used for correcting errors of active sites by the structure preparation process in MOE. After the correction, hydrogens were added and partial charges (Amber12: EHT) were calculated. Energy minimization (AMBER12: EHT, root mean square gradient: 0.100) was performed.

#### Binding site analysis

The binding site of receptors was identified through the MOE. Site Finder program, which uses a geometric approach to calculate putative binding sites in a protein, starting from its tridimensional structure. This method is not based on energy models, but only on alpha spheres, which are a generalization of convex hulls. The prediction of the binding sites, performed by the MOE Site Finder module, confirmed the binding sites defined by the co-crystallized ligands in the holo-forms of the investigated proteins.

#### MOE stepwise docking method

The crystal structures of the enzymes were obtained. Water and inhibitors molecule was removed, and hydrogen atoms were added. The parameters and charges were assigned with MMFF94x force field. After alpha-site spheres were generated using the site finder module of MOE. The optimized 3D structures of molecules were subjected to generate different poses of ligands using the triangular matcher placement method, which generates poses by aligning ligand triplets of atoms on triplets of alpha spheres represented in the receptor site points, a random triplet of alpha sphere center was used to determine the pose during each iteration. The pose generated was rescored using London dG. scoring function. The poses generated were refined with MMFF94x force field, also, the solvation effects were treated. The Born solvation model (GB/VI) was used to calculate the final energy, and the final assigned poses were assigned a score based on the free energy in Kcal/mol. [18].

#### ADMET predictions

The ADMET in silico profile was applied using “MOE” and “admet SAR" tools to the prediction of pharmacokinetic and ADMET characters (absorption, distribution, metabolism, excretion, and toxicity).

### In-vitro studies

#### In-vitro anti-arthritic study

The enzymatic assays of COXs, LOX, TAC, Catalase, MDA, GSH, SOD, TNF -α, and MPO were determined colorimetrically as mentioned previously in reports [19–21].

#### Lymphocyte proliferation activity using MTT reduction assay

The proliferation activity was determined by measuring mitochondrial activity using the MTT reduction method according to Mosaddegh et al*.*, 2012 [22].

#### Statistical analysis

For the assays, linear regression was done for the calculation of IC_50_ (50% inhibitory concentration). Microsoft Excel 2010 program. All the data in Table 4 was represented as mean ± SD [23].

## Results

**Fig. 1:**
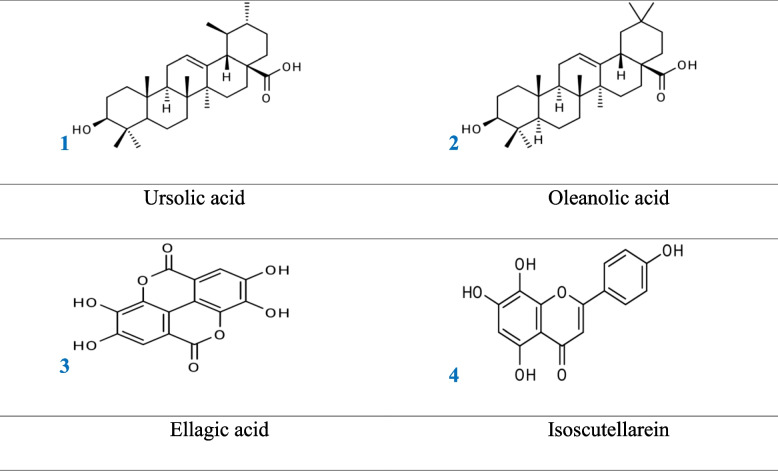
Compounds isolated from aerial parts of *S.m*

All the isolated compounds were obtained from the fraction of ethyl acetate *S.m* aerial parts methanolic extract, twelve compounds were isolated and discovered; eight of them were isolated before by authors [9], and the other isolated four compounds were reported here and identified as follow (Fig. 1).

Ursolic acid (1) The compound was obtained from a silica gel column after further purification as an off-white powder (18 mg), The UV spectrum displayed λ _max_ (MeOH) at 438, and 419 nm and EI-MS gives a peak of molecular ion M^+^ at *m/z* = 456 for this formula C_30_H_48_O_3_. The ^1^H-NMR spectrum was recorded at 600 MHz in CDCl_3_ which gave different signals at d in ppm = 0.73, 0.78, 0.79, 0.93, 0.94, 0.95, and 1.15 (7 s, 21H, due to CH_3_ groups), The other protons have appeared as 1.38 (m, 2H, H-21), 1.39 (m, 2H, H-16), 1.44 (m, 2H, H-20), 1.52 (m, 4H, H-18, H-19, and H-15), 2.10 (m, 3H, H-1, and H-9), 2.14 (m, 2H, H-14), 3.18 (t, 2H, J = 7 Hz, H-2), 3.39 (s, 2H, H-7), 4.57 (s, 2H, H-11), 4.59 (s, 1H, H-12). While the ^13^C-NMR data (150 MHz, CDCl_3_): δ (ppm) at (from C-1 to C-30) 39.3, 28.2, 78.2, 38.3, 55.9, 18.7, 33.8, 40.1, 48.2, 37.6, 23.7, 125.7, 139.4, 42.6, 28.8, 25.0, 48.2, 53.6, 39.5, 39.5, 31.2, 37.5, 28.8, 16.5, 15.6, 17.6, 24.1, 179.7, 17.6, 21.5. all these data were followed and reported for a triterpene compound which was identified as ursolic acid [24].

Oleanolic acid (2) The compound was found as a white powder (24 mg), UV λ _max_ (MeOH) 209 nm. The ^1^H -NMR spectrum (600 MHz, CDCl_3_) proved the presence of the following signals: 0.79, 0.89, 0.91, 0.92, 0.97, 1.08, and 1.34 (7 s, 21H, all-CH_3_), 1.37 (m, 2H, H-21), 1.37 (m, 2H, H-16), 1.52 (m, 5H, H-18, H-19, and H-15), 2.08 (m, 3H, H-1, and H-9), 3.16 (t, 1H, J = 7 Hz, H-2), 3.36 (s, 2H, H-7), 4.58 (s, 2H, H-11), 4.61 (s, 1H, H-12). The ^13^C-NMR spectral data (150 MHz, CDCl_3_): at δ (ppm) were assigned as follows: from C-1 to C-30 appeared at 39.4, 28.4, 78.3, 39.4, 55.9, 18.7, 33.3, 39.9, 48.3, 37.5, 23.8, 122.7, 144.9, 42.8, 28.5, 23.8, 46.8, 42.2, 46.6, 31.2, 34.2, 33.4, 28.8, 16.6, 15.7, 17.6, 26.4, 180.3, 33.4, 23.9. There is a good agreement between the obtained data and the data reported by Werner et al., 2003 [25], for the compound which was identified as oleanolic acid.

Ellagic acid (3) The compound was obtained as a white amorphous powder (20 mg) after multi-purification Sephadex -LH20 column. The UV spectral data λ _max_ (MeOH) 250 nm, the ^1^H-NMR spectrum (600 MHz, CDCl_3_) showed one signal at δ (ppm) at: 7.39 (2H, s, H-4,9) and other signals at 10.53 (s, 4H, -OH groups). The ^13^C-NMR (150 MHz, DMSO-d6) data showed signals at δ (ppm) as 158.5 (C = O, 5, 10), 147.9 (C-3, 8), 139.7(C-2,7), 136.9 (C-1a, 6a), 112.6 (C-4b,9b), 110.9 (C-4, 9), 107.5 (4a, 9a). So, this compound was identified as ellagic acid [26].

Isoscutellarein (4) The compound was found as a yellow powder (25 mg) and it is an aglycone in nature according to its chromatographic behavior on paper in different solvents. The UV spectral data λ_max_(nm) in MeOH: 269, 338 indicate the flavone type of this compound. The bathochromic shift in band-I with increasing intensity on the addition of NaOMe: (255, 401) proves the presence of a free OH group at C-4'; (AlCl_3_): 267, 287, 401; (AlCl_3_/HCl): 267, 307, 355; while the bathochromic shift in band-II (5 nm) on the addition of NaOAc (274, 369) substantiate the occurrence of a group of free OH at C-7 and (NaOAc/H_3_BO_3_): 274,374 [27].

The ^1^H-NMR (600 MHz, DMSO-*d*6,) spectrum displayed signals at δ (ppm)7.62 (2H, d, J = 8.5 Hz, H-2', 6'), 6.81 (2H, d, J = 8.5 Hz, H-3', 5'), The two aromatic singlets at 6.74 and 6.52 were assigned to H-3 and H-6, respectively. The appearance of a single signal at δ 6.62 ppm of H-6 more downfield than normally indicated that the carbon (C-8) is oxygenated. While the ^13^C-NMR (150 MHz, DMSO-d6) at δ in ppm 182.3 (C-4), 163.8 (C-2), 162.3 (C-4), 161 (C-9), 155 (C-5), 161.4 (C-7), 128.9 (C-2, 6), 128.7 (C-8), 121.5 (C-1), 116.8 (C-3, 5), 103.4 (C-10), 103.8 (C-3), 98.7 (C-6). These data supported the identification of compound 4 as isoscutellarein [28].

### Toxicological study in silico

#### Pharmacokinetic profile in silico

**Fig. 2:**
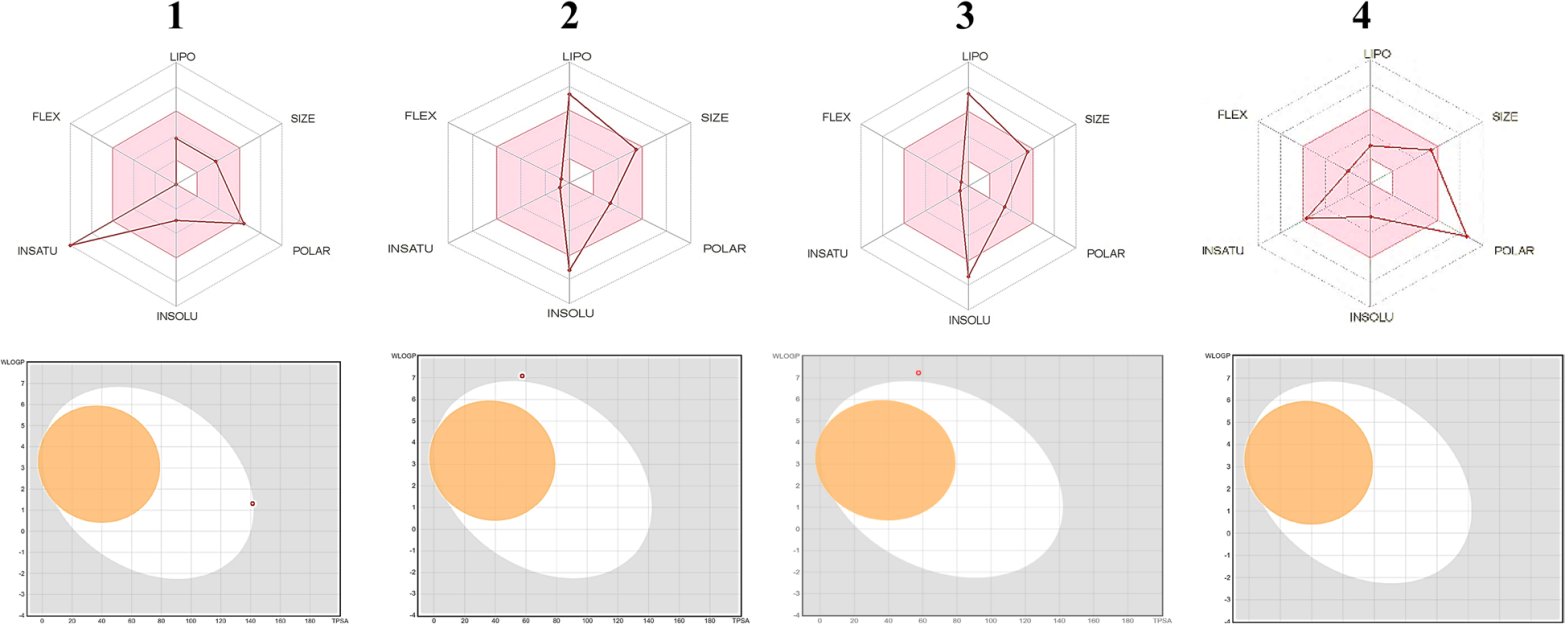
Bioavailability Radar plot of 1–4, respectively. Each attribute's optimum range is represented by the pink area (Lipophilicity: 0.7 XLOGP3 + 5.0, size: 150 MW 500 g/mol, polarity: TPSA 140 2, solubility: log S 6, and flexibility: rotatable bonds 9. The white zone represents very probable HIA (GI) absorption, whereas the yellow zone shows very likely BBB permeability. The outer grey region is made up of compounds that are expected to have poor absorption but no brain penetration. Furthermore, the blue point indicates P-gp substrate (PGP +), whereas the red point shows P-gp non-substrate (PGP-)

In silico cytotoxicity screening utilizing ADMET parameters is a critical step in the creation of therapeutic bioactive compounds. The descriptors for 1–4 were generated using MOE, Swiss ADMET [29], and the admit-SAR model [30], as shown in Table 1. Compounds 1–4's physicochemical and ADMET properties revealed that they met Lipinski's criteria, with molecular weight as an exception. Furthermore, the tested compounds fail when the Ghose Veber, Egan, and Muegge criteria are used. Consequently, the tested compounds exhibited a range of bioavailability scores (0.85- 0.17) with biodegradation range values (0.78 to 0.96). These data revealed that the compounds tested had a high oral bioavailability but little biodegradability. The Bioavailability Radar planner created a lead-likeness profile for the medication under consideration (Fig. 2). Pink denotes the ideal range for each feature (polarity, size, lipophilicity, solubility, saturation, and flexibility).

**Table 1:** ADMET data for compounds 1–4

		**1**	**2**	**3**	**4**
**Absorption**					
Blood–Brain Barrier	BBB +	0.5641	0.7761	0.783861	0.855371
Human Intestinal Absorption	HIA +	0.7199	0.863	0.763	0.8758
Caco-2 Permeability	Caco2-	0.8307	0.8353	0.843653	0.920618
P-glycoprotein Substrate	Substrate	0.5382	0.7801	0.787901	0.859779
P-glycoprotein Inhibitor	Non-inhibitor	0.9377	0.8511	0.859611	0.938031
	Non-inhibitor	0.9639	0.781	0.78881	0.860771
Renal Organic Cation Transporter	Non-inhibitor	0.9307	0.8312	0.839512	0.916099
Subcellular localization	Mitochondria	0.7339	0.8699	0.878599	0.958752
Aqueous solubility		-3.144	-4.3883	-4.43218	-4.83652
Caco-2 Permeability		-0.076	1.5443	1.559743	1.702035
CYP450 2C9 Substrate	Non-substrate	0.8339	0.8258	0.834058	0.910147
CYP450 2D6 Substrate	Non-substrate	0.9096	0.8973	0.906273	0.98895
CYP450 3A4 Substrate	Non-substrate	0.7205	0.7901	0.798001	0.870801
CYP450 1A2 Inhibitor	Non-inhibitor	0.5914	0.9169	0.926069	1.010552
CYP450 2C9 Inhibitor	Non-inhibitor	0.5591	0.9071	0.916171	0.999751
CYP450 2D6 Inhibitor	Non-inhibitor	0.9575	0.9485	0.957985	1.04538
CYP450 2C19 Inhibitor	Non-inhibitor	0.8017	0.9025	0.911525	0.994681
CYP450 3A4 Inhibitor	Non-inhibitor	0.9078	0.8695	0.878195	0.958311
CYP Inhibitory Promiscuity	Low CYP Inhibitory Promiscuity	0.9568	0.9046	0.913646	0.996996
Human Ether-a-go-go-Related Gene Inhibition	Weak inhibitor	0.9721	0.9582	0.967782	1.056071
	Non-inhibitor	0.9152	0.813	0.82113	0.89604
AMES Toxicity	Non-AMES toxic	0.9127	0.849	0.85749	0.935717
Carcinogens	Non-carcinogens	0.9582	0.9394	0.948794	1.03535
Fish Toxicity	High FHMT	0.9615	0.9924	1.002324	1.093764
Tetrahymena Pyriformis Toxicity	High TPT	0.9583	0.985	0.99485	1.085608
Honeybee Toxicity	High HBT	0.5796	0.8437	0.852137	0.929876
Biodegradation	Not readily biodegradable	0.8051	0.962	0.97162	1.060259
Acute Oral Toxicity	II	0.602	0.8316	0.839916	0.91654
Carcinogenicity (Three-class)	Non-required	0.6954	0.5962	0.602162	0.657096
Rat Acute Toxicity		2.6213	2.3902	2.414102	2.634335
Fish Toxicity		0.526	0.8147	0.822847	0.897913
Tetrahymena Pyriformis Toxicity		0.386	0.9588	0.968388	1.056732
**Drug likeness**		0	0	0	0
No. H-bond acceptors		3	3	3	3
No. H-bond donors		2	2	2	2
Fraction Csp3	Saturation fraction	0.9	0.9	0.909	0.991926
Molar reactivity	*MR*	136.91	136.91	138.0165	150.6074
Topological surface area	*TPSA*	57.53	57.53	58.1053	63.40611
Log P	Lipophilicity	3.95	3.95	3.9794	4.342432
Log S	Solubility	-7.23	-7.23	-7.3932	-7.06766
Lipinski No. violations		1	1	1	1
Ghose No. violations		3	3	3	3
Veber No. violations		0	0	0	0
Egan No. violations		1	1	1	1
Muegge No. violations		1	1	1	1
Bioavailability Score		0.85	0.85	0.85	0.853
PAINS #alerts		0	0	0	0
Brenk #alerts		1	1	1	1

#### Prediction of oral toxicity

The possible toxicity was predicted [31] by utilizing data from the Chemical European Biology Laboratory (ChEMBL) database to calculate an estimate of rodent oral toxicity. Furthermore, we determined the median fatal dosages (LD_50_) in rats, and our ligands had a high LD_50_. This database's calculated toxicity is based on the highest endpoints, which comprise thirty-three models (Table 1). The estimated toxicity was divided into different stages of toxicity as “toxicity, toxicological endpoints (mutagenicity, carcinotoxicity, organ toxicity (hepatotoxicity), cytotoxicity and immunotoxicity). Toxicological pathways and toxicity targets thereby, providing an appropriate vision into the probable mode of action including the toxic rejoinder. These compounds 1–4 are categorized as low toxicity classes III and do not include any dangerous components or bind to any toxic objectives. In general, the medicines investigated, exhibited no main health effects, and high oral bioavailability, high BBB transport capacity for rodent toxicity profiles.

#### *In silco* detection of immunomodulatory targets

To identify the biological activity of the isolated compounds we targeted active sites of human immunomodulatory receptors such as NF-kappaB p52 (PDB:1A3Q) at 2.1 A° resolution, Proinflammatory cytokines such as interleukin-1 “IL-1”; (PDB:2NVH [32],) at 1.35 A° resolution, viral interleukin-10 (PDB:1VLK [33]) at 1.09 A° resolution, anti-inflammatory receptor such as cyclooxygenase-2 (PDB:1CX2) at 3.00 A° resolution and antioxidant activity as (PDB:1P9M(at 3.65 A° resolution). The gold score function, which was included in MOE 2021, was used to calculate the behavior of the ligand-protein interaction. All docking experiment calculations were shown in (Table 2). The crystal structures were derived from the protein databank. The ligands under consideration docked into the active site for these enzymes.

**Table 2:** Docking energy scores (Kcal/mol) for isolated compounds (1–4)

No	S	E_MD_	RMSD	E_conf	E_place	E_Int	E_vdw	Interaction	Bond type	Distance	E (Kcal/mol)
**1VLK**											
1	-4.66	-5.21	1.49	8.97	-93.34	-9.32	-23.14	GLY61	π-π	2.76	-0.5
2	-6.13	-6.41	1.69	89.26	-112.64	-8.73	-23.67	GLY61	H-donor	2.90	-1.1
3	-5.79	-6.52	1.33	58.30	-123.27	-10.42	-29.50	GLY58	H-donor	2.96	-0.9
								TYR59	H-donor	3.03	-0.8
								CYS62	H-acceptor	3.06	-2.4
4	-5.89	-6.83	1.57	54.20	-63.46	-10.69	-33.65	PHE15 ASP13	H-donor H-donor	2.88 2.94	-1.8 -1.6
**1A3Q**											
1	-6.15	-6.75	1.27	11.49	-111.71	-14.05	-35.09	Lys283	π-π	2.76	-0.5
2	-2.49	-7.43	1.10	208.93	-122.80	-11.50	34.45	Lys283	π-π	2.90	-0.6
3	-3.42	-8.12	1.62	157.69	-156.48	-11.90	26.80	Lys283	H-donor	2.96	-0.8
4	-8.12	-8.80	1.21	57.64	-93.83	-16.24	-49.02	Ser70	H-donor	2.68	-0.5
**2NVH**											
1	-5.14	-6.42	1.29	8.68	-98.04	-12.88	-26.62	GLU25 PHE133	H-donor H-π	2.23 2.98	-1.4 -0.5
2	-5.01	-6.80	1.01	77.53	-137.22	-10.60	-24.39	GLU25 LYS74 LYS77	H-donor H-acceptor H-acceptor	2.91 2.29 2.37	-2.7 -0.5 -0.7
3	-5.43	-7.19	1.33	66.30	-148.91	-10.26	-25.28	GLU25	H-π	2.23	-0.5
4	-6.28	-7.57	1.18	57.01	-107.40	-10.28	-37.55	LYS74 ASP75	H-donor H-acceptor	3.24 2.94	-0.5 -3
**1CX2**											
1	-5.89	-8.55	1.80	18.67	-28.93	-14.55	-23.26	TYR355	H-acceptor	3.73	-0.1
2	-11.15	-8.67	1.01	235.79	-61.44	-10.15	29.09	ARG120 TYR355	H-acceptor H-acceptor	2.72 2.82	-0.5 -2
3	-12.20	-8.79	1.57	238.29	-47.70	-10.91	49.76	TYR355	H-acceptor	2.73	-1.1
4	-10.41	-8.92	1.13	109.65	-17.23	-10.69	-8.35	Ser530 TYR355	H-donor H-acceptor	2.93 2.7	-1.4 -1.8
**Ref.1**	-8.36	-8.25	1.16	89.13	-15.36	-8.65	-9.48	ARG120	H-acceptor	1.5	-2.69
**1P9M**											
1	-5.21	-7.48	1.86	20.29	-119.92	-4.69	-17.18	Arg168	H-acceptor	2.91	-2.5
2	-6.41	-7.98	1.80	71.47	-121.35	-6.56	-34.99	Lys66	H-acceptor	2.14	-0.5
3	-6.52	-8.47	1.04	55.47	-139.93	-5.97	-38.11	Cys192 Lys66	H-donor H-acceptor	2.62 2.12	-0.7 -0.6
4	-6.83	-8.97	1.46	55.56	-71.64	-1.62	-43.46	SER137 ALA58 PHE134 PHE134	H-donor H-acceptor H-π H-π	3.52 3.9 4.15	-0.8 -0.5 -0.8 -0.7

**S**; The ligand's final free binding energy from a particular posture, **E conf**; The ligand's free binding energy from a particular conformer. **E place**; The ligand's free binding energy from a receptor. **E Int**.: Ligand affinity binding energy with the receptor, Electrostatic interaction with the receptor, EeleVan der Waals energies between the ligand and the receptor are denoted by the abbreviation **Evdw**. **RMSD** is the root mean square deviation of the docking posture from the co-crystal ligand location. **Ref.1**:1-PHENYLSULFONAMIDE-3-TRIFLUOROMETHYL-5-PARABROMO PHENYLPYRAZOLE

The docking protocol was affirmed before estimating docking calculations by the self-docking of identified compounds with targeted enzymes in their original binding pocket *viaX*-ray. The obtained pose was subsequently equated to that of the *X*-ray structures obtained experimentally. For the isolated compounds, the root means square deviation (RMSD) was lower than 2Å**.** This viable docking protocol was then used to expeled docking calculations for the test ligands. Furthermore, calculation of the molecular dynamic as with ordinary procedures using MOE (Table 2). All enzymes by compared with molecular docking displayed more healthier molecular dynamic free energy. In addition, the applied molecular dynamics had no effect on the bond formation.

### Biological study

#### In-vitro anti-inflammatory effect of extracts on (COX-1, COX-2, 5-LOX, and MPO)

To investigate the effects on COX-1, COX-2, and 5-LOX, the plant material was extracted with 80% aqueous methanol. The extract was evaluated for anti-inflammatory activity using COX-1, COX-2, 5-LOX, and MPO inhibition assays, which are the major mediators of inflammation (Table 3). The total extract showed the higher anti-inflammatory potency against reference drug with the lower IC_50_ value for COX-2 (0.17 μg/ml) and LOX (9.25 μg/ml), MPO (9.50 ng/ml), followed by COX-1 (10.21 μg/ml).

**Table 3:** IC_50_ values of the anti-inflammatory activity of *Satureja montana* alcoholic extract

**Code**	**COX1** IC_50_ μg/ml	**COX2** IC_50_ μg/ml	**LOX** IC_50_ μg/ml	**MPO** IC_50_ ng/ml
Celecoxib	15.1	0.049	11.6	
diclofenac sodium	5.29	0.30	-	
Indomethacin	0.041	0.51	-	
Total extract	**10.21**	**0.17**	**9.52**	**9.54**

#### In-vitro antioxidant assay of extracts on (TAC, Catalase, MDA, and SOD)

Total antioxidant capacity is the measure of the number of free radicals scavenged by a test solution, being used to evaluate the antioxidant capacity of biological samples. The assay showed that the lowest IC_50_ values are 10.4 IU/ml and 11.3 ng/ml for SOD and MDA, respectively, followed by Catalase (14.6 IU/ml), then TAC (22.3 IU/ml). The standard ascorbic acid results were (19.7, 17.2 and 13.1 IU/ml) for TAC, Catalase and SOD, respectively.

#### In-vitro apoptotic Assay of extract (GSH and TNFα)

Excessive apoptosis in the testis has been related to Leydig cell damage and a decrease in testosterone levels in the blood. Furthermore, an increase in the death of Leydig cells results in a decrease in testosterone. The apoptotic extract's assays (GSH & TNFα) were done. The extract showed good IC_50_ values of 16.41 pg/ml for TNFα in comparison to 23.1 pg/ml for (GSH). These findings indicate that the extract possesses antiapoptotic properties**.**

#### Cell viability measurement

The MTT assay was performed to evaluate the effect of the extract on cell viability in mouse peritoneal macrophage cells in vitro. At whatever dosage of the treatment, no significant difference in the percent lives cell population was seen as compared to normal cells (Table 4).

**Table 4:** Immuno-modulatory using MTT results, data expressed as mean ± SD

Control	Extract (50 µg/ml)	Extract (20 µg/ml)	Extract (10 µg/ml)	Extract (5 µg/ml)
2.76	0.86	2.47	3.87	2.55
3.24	1.22	2.54	3.65	2.34
2.95	1.65	2.30	2.92	1.87
2.27	0.98	2.22	3.43	1.98
3.21	1.43	2.65	3.95	2.23
Mean ± SD 2.89 ± 0.39	1.23 ± 0.32	2.49 ± 0.22	3.56 ± 0.41	2.19 ± 0.27

## Discussion

The first look of 1–4 molecules showed variations such as saturation with Csp = 0.9 for 1, solubility and lipophilicity for 2 and 3; and polarity for 4, respectively (Fig. 2). Both compounds (2 and 3) exhibited a high saturation degree in the area (Fraction Csp3 = 0.9- 0.29), which allowed them to pass the Fraction Csp30.25 filter [34]. When applied Logs [35], these compounds exhibited excellent solubility in H_2_O, which is a critical factor in absorption. Lipophilicity criteria in medicinal chemistry were also used to detect hazardous regions in bioactive compounds as a lead-likeness filter, depending on two structural warnings known as PAINS and Brenk's filters. No structural warnings for compounds 1–3 against the PAINS filter, but compound 4 showed one alert.

Using admet-sar pharmacokinetic characteristics, mutagenicity, tumorigenicity, reproductive efficacy, irritability, and human intestine absorption were investigated in silico [36]. In addition, the other (SVM) method was used to determine the substrate or non-substrate of skin permeability (Log Kp), Caco-2, blood–brain barrier (BBB), and p-glycoprotein (P-gp), as well as to discover inhibitory influence on the main cytochromes P_450_ isoenzymes (CYP1A2, CYP2C19, CYP2C9, CYP2D6, CYP3A4). The obtained results of (Table 1) indicated that the isolated 1–4 inhibited both CYP2C9 and CYP3A4 but had no inhibitory effect against any other P_450_. The BOILED-Egg model is presented in (Fig. 2) as a connection for WLOGP versus TPSA, indicating that, the human Ether-à-go-go-Related Gene (hERG) against High BBB permeability, high GI absorption, high brain penetration, and ambiguous inhibitory action. Furthermore, the compounds (1–4) presented P-gp activation (multidrug resistance protein 1). Furthermore, negative skin permeability (Kp) values for isolated compounds revealed that all compounds exhibited low skin permeability. The carcinogenic behavior of 1–4 compounds was investigated and compared with 981 other carcinogenic chemical structures selected from the “Carcinogenic Potency Database (CPDB) [37], with the findings indicating that the tested compounds had no carcinogenicity, mutagenicity, or tumorigenicity effects.

The enzymes' active sites were identified as residues within a 3.5 radius of the atoms of reference medications. The molecular docking was carried out with the use of an (AMBER12:EHT) force field with a gradient convergence range of 0.05 kcal/mol, which was utilized to decrease the energy for the produced ligand-enzyme complexes. To evaluate the tested ligand binding affinity, the highest MOE scoring function was employed (Table 2).

### In the case of 1VLK

**Fig. 3:**
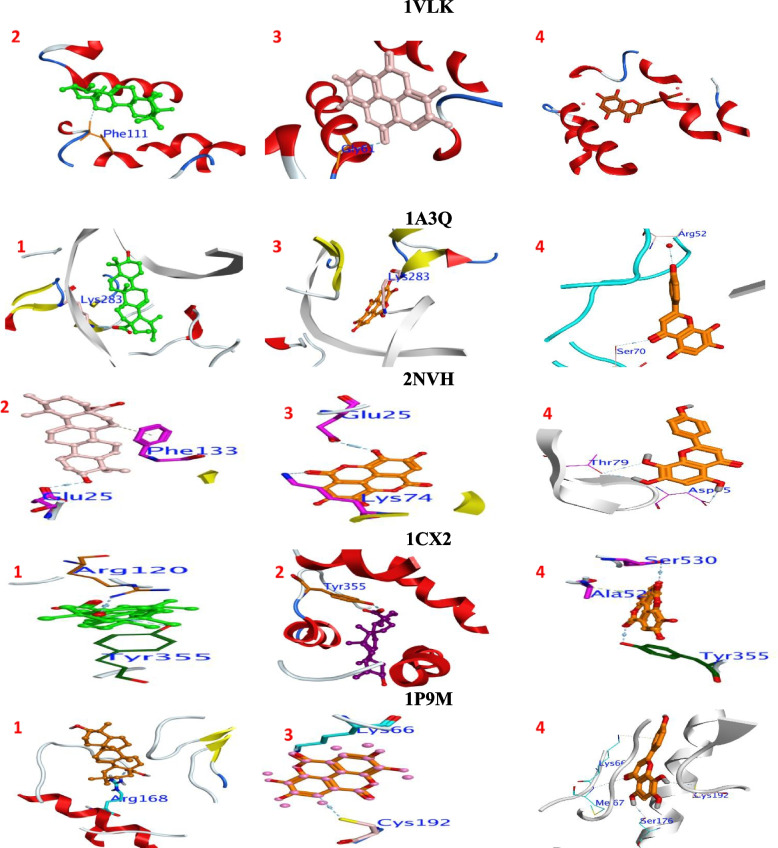
Interaction between the tested compounds (1–4) with binding sites of tested enzymes

The 1–4 ligands revealed MOE score arranged as 2>4>3>1(-6.1, -5.8, -5.7, and -4.6 kcal/mol), respectively (Table 2). The 1–4 hybrids occupied the binding pocket by the formation of H-bond interactions with important amino acids Gly61 and Phe111 (Fig. 3). It was hypothesized that hydrophobicity and membrane permeability are important pharmacokinetic characteristics for absorption compounds in biological systems due to their strong interaction with hydrophobic residues of the binding site.

### In the case of 1A3Q

The binding affinities were arranged as 4>1>3>2 (-8.1, -6.1, -3.4, and -2.4 kcal/mol), respectively (Table 2). These clusters localized in the binding site through H-bond interactions with important amino acids Lys252 & Lys283. The interaction mode of tested compounds with nuclear factor NF-kappa B p52 demonstrated that, the vital role of hydrophilicity in binding interaction.

### In the case of 2NVH

Compound 4 has the highest binding affinity(-6.28 kcal/mole) when compared with all tested compounds 2–4. That showed nearly the same binding scores (-5.4, -5.01, and -5.4 kcal/mol), respectively for 2–3 (Table 2). These compounds formed H-bond interactions with important amino acids Gly25 Lys74 The 79, phe 133, and Gly138.

### In the case of 1CX2

The binding affinities were arranged as 4> 3 > 2>1 These clusters localized in the binding site through H-bond interactions with important amino acids Ala52 Arg120 Tyr355 and Ser530.

### In the case of 1P9M

The 1–4 ligands revealed MOE scores arranged as 4>3>2>1 respectively **(**Table 2). All compounds showed nearly the same binding affinity of about -6 kcal. /mol. The 1–4 hybrids occupied binding pockets by formation H-bond interactions with Lys66, Gln135, Arg168, and Gln 135, Fig. 3.

Inhibition of the remarkable proinflammatory mediators with the plant secondary metabolites are significant in recent years. The mediators act simultaneously, to initiate and endure the inflammatory cascade [38]. From plants, the pure compounds and crude extracts, aim these mediators and flagged the way for evolution of a new therapeutic accession [39]. The high phenolic content of *S. montana,* exhibited reducing power and high free radical-scavenging properties [3, 9].

The in vitro biological activity of *S. montana* alcoholic extract, displayed a good antioxidant and anti-inflammatory activity in addition to apoptotic activity. IC_50_ for COX1 & 2 were 10.21 and 0.17 µg/ml in comparison to 15.1 and 11.6 µg/ml for celecoxib (standard). TAC of the total alcoholic extract in our results was 22.3 IU/ml compared to 19.7 IU/ml of the standard ascorbic acid.

Catalase is a unique enzyme, which protect cells from oxidative damage and play a vital role, with catalyzing the breakdown of hydrogen peroxide into water and oxygen, forming noticed, efficient and specific complex chemical reactions. Catalase IC_50_ value was (14.6 IU/ml), compared to 17,2 IU/ml of the standard ascorbic acid.

Superoxide dismutase is also an enzyme, has a special role in catalyzing the dismutation of superoxide radicals into oxygen and hydrogen peroxide for protecting cells from oxidative stress. In additition to keep normal physiological processes, so its deficiency led to different pathological conditions, such as neurodegenerative disorders, cancer, and aging. SOD IC_50_ value was (10.4 IU/ml), compared to 13,1 IU/ml of the standard ascorbic acid. These results coincide with previous studies that reported, the presence of flavonoids, phenolic acids, alkaloids, and terpenoids in plants modulate SOD activity relying on their concentration and chemical structure [40].

The reduced glutathione is a potent antioxidant, that neutralize ROS, in addition to other free radicals, which produced through metabolic processes. GSH act as a cofactor for different enzymes such as glutathione peroxidase and glutathione S-transferase in detoxification pathways. GSH IC_50_ value was (23.1 pg/ml), which indicating that the extract possesses antiapoptotic properties. These results indicated that *Satureja montana* alcoholic extract possess a remarkable antioxidant effect. In addition, it also lowers MDA level to 11.3ug/ml compared to 15.3 for standard ascorbic acid [41].

## Conclusions

The most common species in the *Satureja* genus is *Satureja montana* (family Lamiaceae). Four recognized substances were extracted and identified from the ethyl acetate fraction of *S. montana* methanolic extract in this investigation. The anti-inflammatory and antioxidant profiles of the alcoholic extract, as well as the molecular docking of the isolated compounds, were investigated, and it was discovered that they had potential anti-inflammatory and antioxidant capabilities. The drug investigations, such as similarity and ADMET, suggested that their ligands may have a favorable pharmacokinetic profile with no carcinogenic impact. The isolated compounds 1–4 were subjected to a molecular docking analysis, which revealed promising binding affinity against all enzymes. As a result, these compounds show promise as antioxidants, anti-inflammatory agents, and anticancer agents. Theoretical investigations were supplemented with experimental data.

## Data Availability

All data generated or analysed during this study are included in this published article.
